# Influence of sociodemographic and clinical factors on illness progression and quality of life among older adults in Ibadan, Nigeria

**DOI:** 10.1186/s12877-025-06458-8

**Published:** 2025-10-21

**Authors:** Oluwayemisi T. Olaoluwa, Mojisola S. Ajayi

**Affiliations:** Department of Psychology, University of Ibadan, Ibadan, Nigeria

**Keywords:** Elderly, Illness cognition, Duration of symptoms, Illness progression, Quality of life

## Abstract

**Background:**

Given the projected increase in the percentage of older adults globally, particularly in highly populated low- and middle-income countries such as Nigeria, there is a pressing need to investigate the role of sociodemographic and clinical factors in the progression of illness and the quality of life of older adults. This study examined the influence of sociodemographic characteristics (age, sex, marital status, educational qualifications, and income status) and clinical factors (duration of symptoms before treatment and illness cognition) on illness progression and quality of life among older adults residing in Ibadan, Nigeria.

**Method:**

A Sequential explanatory mixed-methods research design, which combines quantitative and qualitative approaches, was adopted. The quantitative approach involved a self-reported survey of fifty (50) older adults (65 years and above) using psychometrically sound scales. The qualitative aspect adopted a phenomenological theory utilising key informant interviews and thematic analysis among healthcare professionals for further insights. Both phases of the study were conducted at Jericho Specialist Hospital and the Chief Tony Anenih Geriatric Centre in Ibadan. The study’s hypotheses were tested using two-step hierarchical regression analysis at a 0.05 level of statistical significance.

**Results:**

The findings of the quantitative phase indicate a significant joint influence of clinical factors, such as the duration of symptoms before treatment and illness cognition, on quality of life when perceived social support is controlled for (*R* = .16, F(3, 46) = 2.82, *p* < .05). The results of the explanatory phase of the study identified four themes, which further justify the roles of sociodemographic and clinical factors in influencing the progression of illness and the quality of life of older adults. These themes are the issue of ageing, health motivations, treatment outcomes, and the need for stakeholders’ involvement.

**Conclusion:**

Duration of symptoms before the commencement of treatment and illness cognition play a significant role in the quality of life of older adults. It is recommended that caregivers of older adults and clinicians consider these factors to improve illness progression and quality of life among the elderly. Qualitative inquiry suggests the need for increased public awareness and stakeholder involvement in interventions for the elderly.

## Background

The proportion of the aged in the world population is expected to increase from 12% in 2015 to 22% by 2050. By 2030, 1 in 6 people will likely be over 60 [1]. Nigeria, the most populous nation in Africa with the highest number of older adults on the continent and 19th highest globally, is projected to have an increased proportion of older adults from its yearly constant of 2.8% to 10.1% by 2100 [2]. Older adults experience a combination of several health conditions, including osteoarthritis, depression, diabetes, dementia, hearing loss, and other groups of complex health conditions [1].

The high burden of health problems experienced by the aged can be attributed to the deterioration of health with ageing [3]. Just as chronic conditions occur more with increasing age, the progression of these conditions worsens with age. Illness progression is an individual’s overall well-being as their symptoms develop and illness progresses [4, 5]. Older adults of low socioeconomic status are more likely to have poorer health status and worse illness progression. An individual’s gender also influences the progression of their illness [6]. Individuals with lower educational qualifications have been reported to have worse progression of early-onset Alzheimer’s disease [7]. An older adult’s illness cognition, which means their knowledge, understanding, and perception of their illness, is also a major predictor of the progression of their illness [8].

There is a deterioration in the quality of life of an individual as they age [9]. An older adult’s quality of life can be comprehensively defined as their evaluation of their autonomy, role and activity, relationships, attitude and adaptation, emotional comfort, spirituality, home and neighbourhood, financial security, and perception of their health [10]. This self-assessed quality of life can be influenced by sociodemographic factors such as gender, income [11], and age [12]. Many studies report monthly income as a significant determinant of the quality of life of older adults [11, 13]. Studies report varying effects of education on the quality of life of older adults. A study found that education has a significant relationship with quality of life [14], while another did not report any significant relationship [12]. The marital status of older adult has been reported not to influence their quality of life [14, 15]. These studies point to the importance of investigating the influence of sociodemographic factors on illness progression and quality of life of older adults, especially in low-and middle-income countries like Nigeria. However, there are not enough studies on the influence of both sociodemographic and clinical factors on illness progression and quality of life of older adults.

Studies [16, 17] have shown that the quality of life of an individual, especially one living with a chronic condition, is strongly affected by their illness, cognition and coping mechanisms. An individual’s coping mechanisms are developed based on their illness cognitions. Individuals with positive illness cognition develop protective coping mechanisms, while those with negative illness cognitions are more likely to create negative coping strategies. The direct and indirect influence of these coping mechanisms on an individual’s quality of life makes illness cognition a strong predictor of the quality of life of an older adult [18, 19].

The common-sense model of self-regulation states that individual, external, or illness-related factors influence an individual’s willingness to seek treatment, adherence, and illness progression. For patients with diabetes mellitus, illness cognition influences glycaemic control [17]. According to the Health Belief Model, older adults beliefs about their health condition are a predictor of health-related behaviour, especially their willingness to seek treatment. Illness cognition can be a determinant of when an older adult seeks medical help [20]. These theories suggest that individuals’ clinical factors, like illness cognition and duration of symptoms before treatment, are closely related and major predictors of their illness progression and quality of life. The duration of symptoms before treatment is operationalised in this study as the timeline of seeking medical attention after the initial observable symptoms.

External factors that influence an older adult’s health can be in the form of social support. Older adults with perceived unmet needs have an increase in the progression of illness and require more urgent healthcare compared to those with perceived social support [21]. There has also been a reported significant difference in the physical and mental health of older adults with high perceived social support and those with low perceived social support. Perceived social support, alongside other factors, thus influences illness progression among older adults [22]. Thus, perceived social support can be highlighted as a control variable in the investigation of the influence of sociodemographic and clinical factors on the illness progression and quality of life of older adults.

The selective optimisation with compensation model states that successful ageing involves the maximisation of gains and the minimisation of losses. Losses that often come with ageing are physical and cognitive decline. This points to a relationship between the illness progression of older adults and their quality of life [23]. One of the benefits that can be maximised in aging is social support. Studies have shown a strong and positive relationship between perceived social support and quality of life [24–26]. Research consistently also shows a significant relationship between illness progression and quality of life in older adults, even when perceived social support is controlled for [25, 27, 28].

Despite studies on ageing and quality of life, there is inconclusive information on the role of sociodemographic and clinical factors in illness progression and quality of life among older adults [29]. This study seeks to bridge the gap in information by examining the factors that influence the illness progression and quality of life of older adults. Having established the influence of perceived social support on the variables of concern in this study by multiple studies, it is important to control for the perceived social support of older adults in the investigation of the impact of the independent variables of this study; sociodemographic factors (age, sex, marital status, educational qualifications, and income status) and clinical factors (duration of symptoms duration of symptoms before treatment, and illness cognition) on the dependent variables of illness progression and quality of life of older adults. This study thus investigates the effects of sociodemographic and clinical factors on the illness progression and quality of life among older adults when perceived social support is controlled for.

**Fig. 1:**
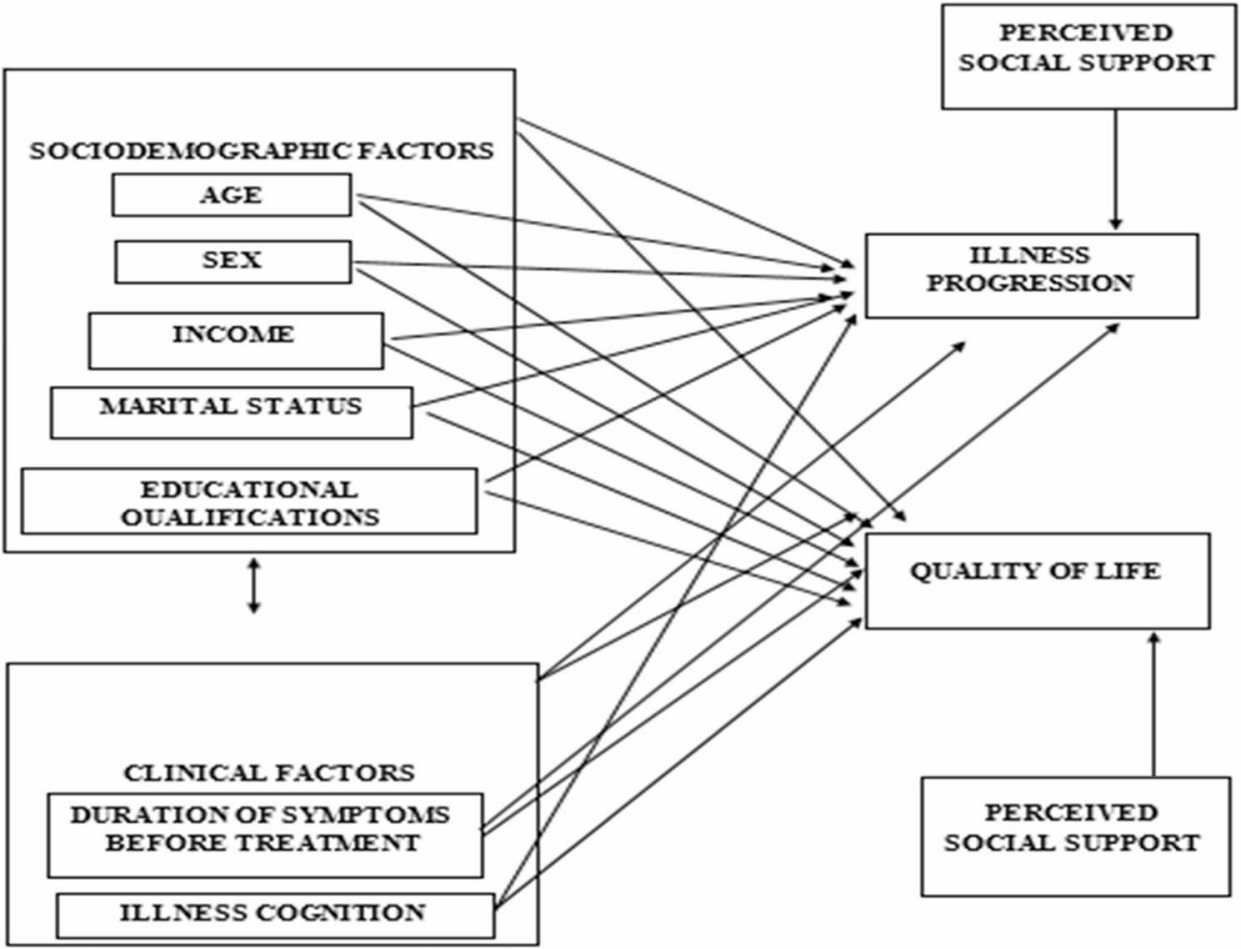
Conceptual Framework *Showing the socio-demographic and clinical conceptual framework of the study as developed by the authors (2025). In this figure*,* a link between the independent variables*,* and the dependent variables is proposed while controlling for perceived social support*

This study provides insights into the factors contributing to the health outcomes of older adults especially in low-and-middle income countries like Nigeria. The study also highlights the importance of examining illness progression and quality of life among older adults and provides scholarly knowledge for geriatric care implementation in different settings (Fig. 1).

## Methods

### Study design

This study uses an explanatory sequential mixed methods design, combining quantitative and qualitative in two phases. The first phase was the quantitative phase which uses a cross-sectional survey design to measure the joint and independent influence of sociodemographic factors, and clinical factors on illness progression and quality of life among older adults while controlling for perceived social support. The explanatory qualitative study was conducted in the form of key informant interviews with healthcare professionals. This study design allows the quantitative investigation of the variables of interest as well as contextual explanation of the results of the quantitative results, especially when they deviate from results of existing studies. The combination of generalizable outcomes with in-depth explanations of these outcomes gives a broader understanding of the variables of interest. It also uses narrative data to explain and complement the quantitative results, especially when there is a small sample size [30, 31].

### Participants and setting

Both phases of the study were conducted at Chief Tony Anenih Geriatric Centre, University College Hospital, the first geriatric centre in sub-Saharan Africa, and Jericho Specialist Hospital, Ibadan. The operational definition for older adults in this study is individuals 65 years and above. The quantitative phase involved 50 persons aged 65 years and above who had been receiving treatment at the outpatient clinics of the Chief Tony Anenih Geriatric Centre, University College Hospital, or Jericho Specialist Hospital in Ibadan. The qualitative phase had six participants, including physiotherapists, nurses, and doctors, who were selected through convenience sampling.

### Quantitative study

The quantitative phase of the study uses a cross-sectional survey design to measure the joint and independent influence of sociodemographic factors and clinical factors on illness progression and quality of life among older adults. Purposive sampling was used to recruit participants who met the specific inclusion criteria relevant to the study. Due to time constraints and challenges in accessing a larger pool of eligible participants, a total of 50 older adults were included without conducting a formal sample size calculation. The inclusion criteria were that participants must be 65 years and above, and most have been receiving treatment at the outpatient clinics of Chief Tony Anenih Geriatric Centre, University College Hospital, Ibadan, and Jericho Specialist Hospital, Ibadan, for at least six months. The six-month treatment criterion is set to ensure that the healtcare professionals can have adequate and comprehensive information on their illness and its progression. Patients who are just presenting to the clinic for diagnosis, inpatients, and those below the age of 65 were excluded from the study.

The independent variables are sociodemographic factors identified as age, sex, income, level of educational qualifications, marital status, and clinical factors (duration of symptoms before treatment, and illness cognition). The dependent variables are illness progression and quality of life. Perceived social support is measured as a control variable. The sociodemographic factors of interest are measured as categorical variables, while the clinical factors, control variables, and dependent variables are measured as continuous variables.

#### Instrument

The quantitative data were collected using an 88-item questionnaire comprising psychometrically sound measures of illness cognitions, social support, illness progression and quality of life, which the researcher and research assistants administered. The duration of symptoms before treatment was measured using the time duration (in weeks) between the reported onset of illness and the time of diagnosis. Illness cognition was measured using the 18-item Illness Cognition Questionnaire [32], and perceived social support was measured with the 34-item Perceived Social Support scale for older adults [33]. Illness progression was measured with a 4-item scale developed by the researchers, while the quality of life is measured using the 19-item control, autonomy, self-realisation, and pleasure (CASP-19) scale [34]. The reliability of the scales in the instrument used for this study ranged from 0.66 to 0.80 [32–34]. The instrument was translated into Yoruba for participants who prefer responding in their native language. To achieve this, the instrument was content validated and translated to Yoruba through the forward and backwards translation process by Yoruba language experts [35].

#### Data analysis

The quantitative data were analysed using SPSS version 28, and the hypotheses of the study were tested using a two-step hierarchical regression analysis with a 0.05 level of significance.

### Qualitative phase

The second phase was a qualitative descriptive approach with thematic analysis. The qualitative phase was conducted through key informant interviews with healthcare professionals. This qualitative approach allowed participants to contextualise the quantitative results. There were six participants, consisting of physiotherapists (2), nurses (2), and doctors (2), selected through convenience sampling. The participants were chosen due to their status as healthcare providers for the participants of the quantitative phase of the study and their ability to provide further insights into the population, especially on the difficulty of assessing illness progression and quality of life. The qualitative data were collected using a semi-structured interview guide developed by the researchers for this study [36]. The interview guide consisted of open-ended questions designed to explore illness progression, quality of life, the role of sociodemographic characteristics, duration of symptoms before treatment, and illness cognition. Questions were formulated based on the health belief model [20] and the common-sense model of self-regulation [16]. The guide was developed by the first author and reviewed by the second author. The interview guide contained fourteen (14) questions. The interviews were transcribed and analysed using thematic analysis to identify patterns and themes related to the quantitative findings. The qualitative phase was guided by the common-sense model of self-regulation as a theoretical framework.

#### Ethical considerations

The researcher started this study by submitting a proposal for ethical approval to the UI/UCH Research Ethics Committee and the Oyo State Research Ethics Review Committee. The study was assigned the number UI/EC/24/0065 by the UI/UCH Ethics Committee and AD 13/479/759B by the Oyo State Research Ethics Review Committee. After due ethical approval, the researcher applied for permission to conduct the study in the two data collection centres – Chief Tony Anenih Geriatric Centre, University College Hospital, and Jericho Specialist Hospital, Ibadan. Permission to conduct a research study at Chief Tony Anenih Geriatric Centre was obtained through the Chairman, Medical Advisory Committee (CMAC), Hospital Services Department (HSD), and the Director, Chief Tony Anenih Geriatric Centre. The respondents were duly informed about the research and the guarantee of confidentiality. Written and verbal informed consent was obtained from respondents who indicated interest in participating in the research.

## Results: quantitative study

The summary of sociodemographic characteristics in Table 1 shows that the number of participants is split equally between Jericho Specialist Hospital and Chief Anenih Geriatric Centre. Twenty-eight participants (56.0%) responded in the Yoruba language, while twenty-two (44%) responded using the English language.

**Table 1:** Summary of characteristics of the participants

Variable	*N*	%	Mean	Sd
Hospital				
Chief Tony Anenih Geriatric Centre, UCH	25	50.0		
Jericho Specialist Hospital	25	50.0		
Language				
Yoruba	28	56.0		
English	22	44.0		
Sex				
Male	18	36.0		
Female	32	64.0		
Income Per Month				
Below #50,000	6	12.0		
Between #50,000 To #100,000	17	34.0		
Between #100,001 To #200,000	13	26.0		
Between #200,001 To #500,000	10	20.0		
Above #500,001	4	8.0		
Marital Status				
Divorced	1	2.0		
Married	27	54.0		
Widow/Widower	22	44.0		
Ethnicity				
Benin	1	2.0		
Hausa	2	4.0		
Igbira	1	2.0		
Igbo	2	4.0		
Ijaw	1	2.0		
Esan	1	2.0		
Yoruba	42	84.0		
Educational Qualifications				
No Formal Education	10	20.0		
Primary School Education	11	22.0		
Secondary School Education	11	22.0		
OND/NCE	3	6.0		
HND	5	10.0		
BSc	5	10.0		
MSc	4	8.0		
PhD	1	2.0		
Age (in years)	**-**	**-**	72.56	6.43
Duration of symptoms before treatment (in weeks)	**-**	**-**	253.08	537.07

The mean age of the participants was 72.56 years, with a standard deviation of 6.43 years. On average, the participants had symptoms that lasted 253.08 weeks before treatment, with a standard deviation of 537.07. The duration of symptoms before treatment showed a strong positively skewed distribution with a skewness of 3.295. The Anker monthly living income for Nigeria in 2024 was reported as NGN 307,691 [37]. Only 28% of the participants were earning a family monthly income that could cover their living expenses comfortably based on the Anker monthly living income.

### Hypotheses testing

Hypothesis 1a, which states that sociodemographic factors such as age, sex, marital status, educational qualification, and income status will have a significant joint and independent influence on illness progression of older adults in Ibadan when perceived social support is controlled for, was tested using hierarchical regression analysis. The results of the two-step hierarchical regression analysis are shown in Table 2.

**Table 2:** Summary of hierarchical regression analysis, showing the joint and independent influence of sociodemographic factors such as age, sex, marital status, educational qualification, and income status on illness progression

	Factor	Β	T	*P*	*R*	*R* ^2^	F	*P*
*Model a*	Perceived Social Support	− 0.12	0.84	> 0.05	0.12	0.01	0.70	> 0.05
*Model b*	Perceived Social Support	− 0.16	−1.01	> 0.05				
	Age	0.03	0.19	> 0.05	0.26	0.07	0.51	> 0.05
	Sex	0.17	1.06	> 0.05				
	Income	− 0.14	− 0.94	> 0.05				
	Marital Status	− 0.09	− 0.56	> 0.05				
	Educational Qualifications	0.13	0.86	> 0.05				

The first step of the regression analysis included perceived social support as a predictor. The B-value of Model A was − 0.12, indicating an inverse relationship between perceived social support and illness progression. The results showed perceived social support did not significantly predict illness progression among older adults (R^2^ = 0.01; F _(1, 48)_ = 0.70; *p* >.05). Model b, with the identified sociodemographic factors (age, sex, income, marital status, and educational qualifications) had an R-value of 0.26 and an R^2^ change of 0.07, thus only 7% of the variation in illness progression was accounted for by the independent variables. The change in R^2^ was also found not to be significant either jointly or independently (R^2^ = 0.07; F _(6, 43)_ = 0.51; *p* >.05). Thus, the predictors could not significantly predict illness progression among older adults. The results highlight that when perceived social support is controlled for, sociodemographic factors such as age, gender, marital status, educational qualifications, and income status do not predict illness progression either independently or jointly. The alternate hypothesis is therefore rejected.

Hypothesis 1b, which states that sociodemographic factors such as age, sex, marital status, educational qualification, and income status will have a significant joint and independent influence on the quality of life of older adults in Ibadan when perceived social support is controlled for, was tested using hierarchical regression analysis. The results of the hierarchical regression analysis testing hypothesis 1b are shown in Table 3.

**Table 3:** Summary of hierarchical regression analysis, showing the joint and independent influence of sociodemographic factors such as age, sex, marital status, educational qualification, and income status on quality of life

	Factor	Β	T	*P*	*R*	*R* ^2^	F	*P*
*Model a*	Perceived Social Support	0.33	2.46	< 0.05	0.33	0.11	6.03	< 0.05
*Model b*	Perceived Social Support	0.13	2.34	< 0.05				
	Age	0.223	1.24	> 0.05				
	Sex	2.03	0.86	> 0.05	0.48	0.23	2.12	> 0.05
	Income	0.15	0.15	> 0.05				
	Marital Status	−5.21	−2.41	< 0.05				
	Educational Qualifications	0.40	0.74	> 0.05				

The Model A with perceived social support as a predictor was significant (R^2^ = 0.11; F _(1, 48)_ = 6.03; *p* >.05) and accounted for 11% of the variance in quality of life. The B coefficient shows a positive relationship between perceived social support and quality of life. Perceived social support thus had a significant direct or positive influence on the quality of life among older adults. The sociodemographic factors were added to Model B in the second step of the analysis. Only marital status was a significant independent predictor of quality of life among older adults (β = −5.21; t = −2.41; *p* <.05). Sociodemographic factors were however found not to have a joint statistically significant influence on the quality of life among older adults (R^2^ = 0.48; F _(6, 43)_ = 2.12; *p* >.05). The results highlight that when perceived social support is controlled for, sociodemographic factors such as age, sex, marital status, educational qualifications, and income status do not jointly ort independently predict the quality of life among older adults. Marital status, however, had a significant independent influence on the quality of life among older adults. The alternate hypothesis is therefore partially accepted.

The results of testing hypotheses 1a and 1b show that sociodemographic factors such as age, sex, income, marital status, and educational qualifications do not significantly influence either illness progression or quality of life among older adults. Only marital status was shown to significantly influence the quality of life of older adults.

Hypothesis 2a stated that there will be joint and independent influence of clinical factors such as duration of symptoms before treatment, and illness cognition on illness progression among older adults when perceived social support is controlled for. The hypothesis was examined using hierarchical regression analysis. The results are presented in Table 4.

**Table 4:** Summary of hierarchical regression analysis, showing the joint and independent influence of sociodemographic factors such as duration of symptoms before treatment, and illness cognition on illness progression among older adults

	Factor	Β	T	*P*	*R*	*R* ^2^	F	*P*
*Model a*	Perceived Social Support	.−0.12	− 0.84	> 0.05	0.12	0.01	0.70	> 0.05
*Model b*	Perceived Social Support	− 0.12	− 0.84	> 0.05				
	Duration of symptoms before treatment	0.26	1.85	> 0.05	0.31	0.10	1.63	> 0.05
	Illness Cognition	− 0.12	− 0.89	> 0.05				

The results of the first step of the hierarchical regression analysis indicate that perceived social support is not a significant predictor of illness progression among older adults (R^2^ = 0.01; F _(1, 48)_ = 0.70; *p* >.05). The adjusted R^2^ change value shows that only 1% of the variance in illness progression was accounted for by perceived social support. The second model contained clinical factors (duration of symptoms before treatment, and illness cognition) and was found not to significantly predict illness progression among older adults (R^2^ = 0.10; F _(6, 43)_ = 1.63; *p* >.05). The results highlight that, when perceived social support is controlled for, clinical factors such as duration of symptoms before treatment, and illness cognition do not jointly or independently predict illness progression among older adult. The alternate hypothesis is therefore rejected.

Hypothesis 2b stated that there will be joint and independent influence of clinical factors such as duration of symptoms before treatment, and illness cognition on quality of life among older adults when perceived social support is controlled for. The hypothesis was examined using hierarchical regression analysis. The results are presented in Table 5.

**Table 5:** Summary of hierarchical regression analysis, showing the joint and independent influence of clinical factors such as duration of symptoms before treatment, and illness cognition on quality of life of older adults

	Factor	Β	T	*P*	*R*	*R* ^2^	F	*P*
*Model a*	Perceived Social Support	0.33	2.46	< 0.05	0.33	0.11	6.03	< 0.05
*Model b*	Perceived Social Support	0.34	2.50	< 0.05				
	Duration of symptoms before treatment	0.05	0.36	> 0.05	0.39	0.16	2.82	< 0.05
	Illness Cognition	− 0.21	−1.51	> 0.05				

The results showed that the first model containing the clinical factors (duration of symptoms before treatment, and illness cognition) significantly predict the quality of life among older adults (R^2^ = 0.11; F _(2, 47)_ = 6.03; *p* <.05). The results indicated that model b with perceived social support has statistical significance suggesting that model b is a statistically significant predictor of the quality of life of older adults (R^2^ = 0.16; F _(3, 46)_ = 2.82; *p* <.05). Clinical factors (duration of symptoms before treatment, and illness cognition) have a significant joint influence on quality of life of older adults when perceived social support is controlled for, but have no significant independent influence on the quality of life of older adults. Thus, the alternate hypothesis is partially accepted.

The results of hypotheses 2a and 2b highlight that while the identified clinical factors (duration of symptoms before treatment, and illness cognition) have a significant joint influence on quality of life, the variables do not have a significant joint influence on illness progression among older adults. The variables were also found not to have a significant independent influence on either the illness progression or the quality of life of older adults.

### Qualitative study

From Table 6, it is shown that there were two male respondents and four female respondents. There were also two medical doctors, two physiotherapists, and two nurses. The average years of professional experience was found to be 12.17 (approximately 12 years). Three of the participants were from Jericho Specialist Hospital (JSH), Ibadan, while the other three were from Chief Tony Anenih Geriatric Centre (CTAGC), University College Hospital, Ibadan.

**Table 6:** Summary of participants’ profile

Identifier	Gender	Profession	Years of professional experience	Work Title	Settings
P1	Male	Nursing	4 years	Senior Nursing Officer	JSH
P2	Female	Physiotherapist	23 years	Deputy Director of Physiotherapy	JSH
P3	Female	Medical Doctor	14 years	Consultant Family Physician	JSH
P4	Female	Nursing	16 years	Senior Nursing Officer	CTAGC
P5	Female	Physiotherapy	11 years	Principal Physiotherapist	CTAGC
P6	Male	Medical doctor	5 years	Medical Officer	CTAGC

### Thematic analysis

Three key themes were derived from the analysis of the qualitative study. The themes are discussed below:
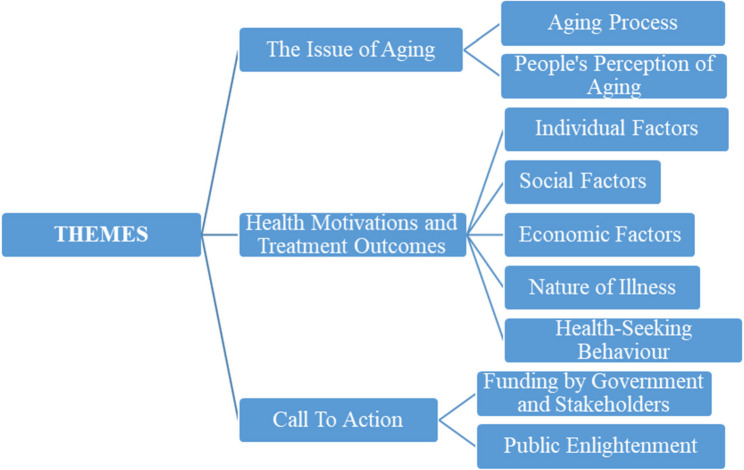


#### Key issue 1: the issue of ageing

This theme describes the participants’ opinions about the ageing process and how it influences the health and quality of life of older adults. The ageing process and people’s perception of ageing were two sub-themes identified from the participants’ comments. Certain bodily changes that occur in an older adult were described to contribute significantly to their illness progression and quality of life. According to the findings of this study, reduced immunity, changes in eating habits, bodily changes, and degenerative changes are some of the changes associated with ageing.

*” As an individual progresses in age*,* they are more prone to some illnesses due to the ageing process. Eating habits also change with age*,* and this lowers immunity in older adults”.* P1.

According to another participant, P5, “*Most of the conditions experienced by the patients we attend to are age-related and incurable*,* so we manage conservatively*,* and this depends on how the patient presents for management*”.

How people perceive their ageing and the changes associated influence their health-seeking behaviour, health outcomes, and quality of life. The study reveals that perceptions of ageing and individual health beliefs significantly influence health-seeking behaviours and illness progression. Participants expressed that aged individuals often view their health issues as a natural part of ageing, leading to a reluctance to seek treatment. The result of this health-seeking behaviour is an increased duration of symptoms before treatment, as well as sociodemographic factors having less influence on their illness progression. The ageing process and people’s perception of ageing was highlighted as major contributors to the reduced influence of sociodemographic factors and clinical factors on illness progression and quality of life of older adults.

#### Key issue 2: health motivations and treatment outcomes

The data indicate that individual factors such as acceptance of illness condition, pre-illness condition, lifestyle, sex, educational qualifications, nutritional status, personality traits, and marital status are sub-themes of health motivations and treatment outcomes, which significantly influence illness progression and quality of life among older adults.

“*Their lifestyle and experiences before the illness determine how the illness progresses. Those who take their health seriously in their youth require less intervention. In the long run*,* those with a good perspective about their health have a better prognosis.”* P1 stated.

According to P5, “*An educated person can search online for more information on their illness*,* while an uneducated person is likely to believe it is a spiritual attack. Individuals’ health beliefs play a role as well*,* but generally*,* educated people seek more medical treatment.*

Participants also revealed the role of marital status and social support.

*“Social support is very important in adherence to medical instructions and progression in a patient’s illness condition*,* especially for those with spouses. Illness progression can be improved with social support*,* patient education and understanding of their illness*.

Economic factors like family income were also indicated to play a role in the illness progression and quality of life among older adults.

“*I work in a secondary health centre and the type of people we see are of low-income status. Most of these people have low social support*,* fast illness progression and a lower quality of life”.* P3.

The nature of the illness older adults are suffering from was also reported to predict their illness progression and quality of life.

*“Some conditions*,* such as terminal illnesses*,* require palliative care*,* while some degenerative conditions*,* like Alzheimer’s and Arthritis*,* can be managed to reduce progression” P2*.

The participants also stated health-seeking behaviour as a factor of interest for the study.

According to P5, *“Their health-seeking behaviour will impact illness progression. Some conditions are managed and influenced by health-seeking behaviour. Even though there is literature on the illness progression of certain conditions*,* an individual’s health-seeking behaviour plays a huge role in their illness progression. The health beliefs of an individual make them seek care faster*,* thereby reducing the progression of illness and increasing the quality of life.*

Despite the influence of other sociodemographic factors like age, sex, income status, and education on illness progression and the quality of life of older adults, the participants’ responses suggest that social support acts as a stronger predictor of illness progression and quality of life. This explains the quantitative results, which shows that sociodemographic factors do not have a significant joint or independent influence on the illness progression of older adults.

The participants’ explanation that individuals with spouses were reported to have better health outcomes compared to other older adults is also in tandem with the results of the quantitative analysis, which shows that marital status is the only sociodemographic factor with a significant independent influence on the quality of life of adults.

#### Key issue 3: call to action

The participants made a call to action to the government and relevant stakeholders for public awareness and funding to ensure better treatment outcomes for older adults.

P6 stated, *“Health education is a way that the illness progression and quality of life of older adults can be improved”*, while P1 highlighted the need for the provision of more equipment and facilities:

*“The unavailability of diagnostic equipment in the hospital also hinders effective diagnosis. There is a need for the intervention of the government and stakeholders to provide these to ensure faster and proper diagnosis.*

## Discussion

The main focus of this study is to examine the influence of sociodemographic factors such as age, sex, marital status, income status, and educational qualifications, and clinical factors, such as duration of symptoms before treatment, and illness cognition, on illness progression and quality of life among older adults in Ibadan.

Sociodemographic factors of age, sex, marital status, educational qualification, and income status did not account for illness progression among older adults in Ibadan, having controlled for perceived social support. This outcome is in contrast to the findings from previous studies conducted among older adults [22, 38]. The contrary results obtained in this study can be attributed to the level of awareness about the ageing process and accessibility to treatment facilities. As stated by participants during the qualitative phase, the ageing process and the perceptions of people to ageing play a critical role in their illness progression and may therefore be responsible for the lack of significance observed with the sociodemographic factors. In addition, this outcome can be explained using the selective optimization with compensation model, which states that older adults use strategies of selection, optimization, and compensation to maintain balance despite reduced resources. Thus, when sociodemographic factors pose a challenge to an older adult’s health status, they will compensate using other means, like lifestyle changes, to maintain optimal well-being [23].

Sociodemographic factors did not jointly predict quality of life among older adults. This contradicts the expectation that sociodemographic factors would account for quality of life outcomes among older adults and negates findings where age, perceived social support, gender, economic status, and educational level were predictors of the quality of life of older adults [25, 38–40]. However, marital status was the sole predictor of quality of life among older adults in Ibadan, having controlled for perceived social support. The explanatory qualitative report supports the quantitative findings and suggests that married older adults have a better quality of life due to the presence of companionship and support. Older adults who regularly attend the hospital for treatment also have health motivations that defy their sociodemographic factors. Thus, this study findings highlight the need for more supportive interventions and public awareness on quality of life among older adults.

The investigated clinical factors (duration of symptoms before treatment and illness cognition) did not influence the illness progression of older adults in Ibadan. Findings contradict past research and models where duration of symptoms and illness cognition affected illness progression. For instance, in a study conducted among patients with human brucellosis, the duration between illness and treatment significantly accelerated illness progression [41]. Similarly, the common-sense model of self-regulation suggests that older adults with illness will develop self-management skills in response to the health threats they experience. Some of the coping skills developed are illness-focused and directed towards improving their illness progression. The theory thus suggests that illness progression is associated with and influenced by the illness cognition of the older adult [42]. The explanatory qualitative findings may provide explanations for the insignificant result obtained in the present study, as other factors such as pre-illness condition, lifestyle and experiences of the participants were identified as playing an important role in their illness progression when compared to the study’s identified variables.

It was observed that clinical factors (duration of symptoms before treatment, and illness cognition) combined to influence the quality of life of older adults in Ibadan. The result is in concordance with previous investigations on quality of life and perception of illness cognition [43]. When older adults hold favourable illness cognitions, they experience better quality of life. Also, the common-sense model of self-regulation provides a plausible explanation for this observed outcome, specifically, stating that people respond to their illnesses using emotional and behavioural cognitive processes. Their perception of their illness guides their coping behaviours, which in turn influence their quality of life [43, 44].

### Limitations

This study was limited by a dearth of literature on the variables of interest especially among older adults within the Nigerian context. The lack of adequate culturally relevant literature also meant that the instruments used had not been standardised for the population. Due to the sensitivity of the population and the timeline of the project, there was no pilot study conducted to revalidate the instruments used. However, the internal consistency (Cronbach’s alpha coefficient) of the tool was assessed. The sensitivity and specificity of the topic also meant that an identifier (the case note number of the older adult) had to be collected for easy identification and response by the healthcare professional assessing their illness progression. This significantly reduced the sample size, as many participants were not willing to share such information.

The timeline of the research was also a major limitation, as only older adults who have been presenting for treatment for over six (6) months were eligible for the study to ensure that their illness progression can be assessed, thus, the sample size was further limited.

### Implications

The study findings highlight the need for more focus on the assessment and management of illness progression among older adults using a multidisciplinary approach. More collaborations must exist between different healthcare providers attending to older adults to ensure a synergised healthcare provision. This will significantly improve the health outcomes of older adults.

The findings suggest the need for increased focus on the psychoeducation of older adults for improved treatment outcomes. The perception of older adults on their aging needs to be addressed to prevent it from harming their health. The influence of ageism and the perceptions of ageing among older adults has been highlighted to be a major contributor to their illness progression and quality of life of older adults. Healthcare professionals need to consistently educate older adults to ensure adherence to medical instructions for better progression of their illness and improved quality of life.

This study also highlights that geriatric healthcare in Nigeria needs more investment in terms of improved facilities, better policy implementation, and training for healthcare professionals. Older adults, regardless of their sociodemographic factors, continue to be disadvantaged in the quality of healthcare provision and the outcomes of the services they receive. Key stakeholders in the government must implement policies that ensure adequate facilities for older adults, improved access to these facilities, and training for healthcare professionals to increase the quality of treatment.

The perceptions of people regarding the ageing process remains negative and this points to a need for more targeted public awareness to debunk myths and foster improved health outcomes of older adults.

## Conclusion

Sociodemographic factors and clinical factors were found to be good predictors of quality of life. Specifically, Marital status, duration of symptoms before treatment and illness cognitions accounted for better quality of life. However, these factors did not account for illness progression among older adults. From the qualitative inquiry, it was revealed that other factors such as pre-illness condition and nature of illness, perception of aging and older adult’s lifestyle choices may play more important role in the illness progression and quality of life of older adults. Beyond sociodemographic factors, the perception and the satisfaction of social support received by older adults was a strong correlate of illness progression and quality of life which was adequately controlled for in the study.

The results of this study highlight the importance of some sociodemographic and clinical factors in explain illness progression and quality of life. However, there is a strong need to consider other factors that are germane and will provide a wholistic perspective about the illness progression and quality of life among older adults. Although, some of the variables identified did not account for a direct influence on illness progression their role as intervening variables and that of other plausible variables as may be considered in future studies.

### Recommendations

This study findings highlight the need for a more comprehensive approach to the treatment of older adults. Healthcare providers should ensure that older adults, regardless of their sociodemographic factors, have social support, positive illness cognition, and coping mechanisms. Education and awareness of the general populace on positive health behaviours is also recommended to further increase awareness. This would ensure that older adults and their caregivers will be properly educated on the factors that influence their illness progression and quality of life, and how to improve them.

While the study’s identified variables may not have a significant influence on illness progression, they may be intervening variables in the influence of other variables. Future studies should also be conducted to investigate the different factors that influence illness progression among older adults. Specifically, the influence of pre-illness condition and the nature of diagnosis should be explored. Young adults should cultivate healthy behaviours to improve their health outcomes as ageing progresses. Older adults should also be encouraged to seek early treatment and develop positive illness cognition, as these have a significant influence on their quality of life.

By implementing these recommendations, it is expected that there will be a more in-depth understanding of the illness progression and quality of life among older adults. It is expected that this understanding will translate to better ageing, better treatment outcomes and improved quality of life for older adults.

## Data Availability

The datasets used and/or analysed during the current study are available from the corresponding author on reasonable request.
